# Shoulder Injuries and Sport-Specific Shoulder Adaptations in Judo Athletes: A Systematic Review

**DOI:** 10.3390/jcm15208020

**Published:** 2026-10-16

**Authors:** Krzysztof Kasicki, Wiesław Błach

**Affiliations:** 1 Department of Sport Theory and Motor Skills, Institute of Sports Sciences, University of Physical Culture in Kraków, 31-571 Krakow, Poland; 2 Faculty of Sport, Wroclaw University of Health and Sport Sciences, 51-612 Wroclaw, Poland; wieslaw.blach@awf.wroc.pl

**Keywords:** shoulder complex, combat sports, glenohumeral joint, shoulder injuries, injury epidemiology, isokinetic strength, range of motion, adaptations

## Abstract

**Background/Objectives:** The shoulder girdle is a frequent injury location in judo, and sport-specific shoulder-complex adaptations have been described in overhead sports, but these areas have not been synthesised for judo athletes. We synthesised evidence on (Q1) the frequency, anatomical profile, type and mechanism of shoulder girdle injuries and (Q2) the structural and functional characteristics of the shoulder complex in judokas. **Methods:** PubMed, Web of Science, Scopus and EBSCOhost were searched up to 7 August 2026. Two reviewers independently selected studies and extracted data. Risk of bias was assessed with JBI tools and ROBINS-I, and certainty of evidence with GRADE. Because injury definitions and measurement protocols were heterogeneous, a narrative synthesis (SWiM) with stratified descriptive analyses replaced the planned meta-analysis. **Results:** Twenty-three reports (22 studies) were included. Across 11 studies, shoulder girdle injuries accounted for 10.2–25.5% of recorded injuries (median 17.1%); this proportion depended on injury definition, exposure context and ascertainment and does not estimate prevalence. The shoulder ranked first or second among anatomical locations in 9 of 11 studies. Acromioclavicular joint injuries and glenohumeral dislocation/instability predominated. In one controlled study, judokas had higher internal rotator strength and a lower external-to-internal rotation (ER:IR) strength ratio than non-athletes; inter-limb asymmetry was inconsistent, and glenohumeral internal rotation deficit was not studied. Certainty of evidence was very low for all outcomes. **Conclusions:** The shoulder girdle is consistently among the most frequently injured locations in judo, whereas evidence for a judo-specific shoulder adaptation profile is sparse, heterogeneous and of very low certainty.

## 1. Introduction

Judo is one of the most widely practised combat sports. The International Judo Federation comprises 205 national federations in five continental unions [1], and the European Judo Union has 51 member federations [2]. Judo has been part of the Olympic programme since Tokyo 1964 for men and since Barcelona 1992 for women [1]. A bout comprises the contest for the grip (kumi-kata), in which the upper limbs act as the pulling (hikite) and lifting (tsurite) hands, followed by attempts to unbalance the opponent and execute a throw (nage-waza), and groundwork (ne-waza). The shoulder complex is therefore loaded repeatedly while gripping, pulling, executing and receiving throws, and controlling falls (ukemi), a loading profile that differs from that of most other contact sports. Improper body positioning during a throw or a fall may expose the shoulder girdle to acute injury, whereas repeated loading may contribute to chronic complaints.

Biomechanical studies have begun to quantify these loads. Video analyses of the thrown athlete (uke) showed peak shoulder accelerations of approximately 68–72 m/s^2^ and velocities of 4.5–5.1 m/s at impact, with a higher velocity in tai-otoshi than in morote-seoi-nage [3] and in tsurikomi-goshi than in sode-tsurikomi-goshi [4]. Inertial sensors have described body-segment accelerations during ukemi as markers of skill and of technique harmfulness [5], hand–mat contact timing was related to head acceleration during backward breakfalls [6], and whole-body accelerometry has quantified contest intensity across weight divisions [7]. Although small and laboratory-based, these studies suggest that throw type, the athlete’s role (tori or uke) and fall control modify shoulder loading, a plausible but untested link with injury risk.

The injury burden of judo is well documented. A systematic review covering the 2008 and 2012 Olympic Games reported an injury risk of 11–12%, with the knee, shoulder and fingers among the most frequently injured sites [8]. Across 7712 athlete-exposures at three consecutive Olympic Games, judo had an injury incidence of 9.6 per 1000 min of exposure, the highest among the Olympic combat sports analysed [9]. Over 21 seasons of French Judo Federation competitions, 3511 injuries were recorded among 316,203 judokas competing in 421,670 bouts [10]. A substantial injury burden has also been reported in national teams [11], among current and former athletes [12], at the European Championships [13] and among university athletes, 61% of whom reported at least one injury within one year [14].

The shoulder girdle typically ranks high in the anatomical distribution of these injuries. In the European Judo Union registry of 128 top-level events held between 2005 and 2020, the shoulder was the second most frequently injured location after the knee, accounting for 15.7% of all injuries [15]. In the French registry, the acromioclavicular (AC) joint was the most frequently sprained structure and the shoulder the most frequent site of dislocation; clavicle fractures predominated in children, whereas glenohumeral dislocations were more common in adults [10]. The clinical consequences can be serious, as illustrated by surgical series of recurrent anterior glenohumeral instability and return to competition in judokas [16,17].

In parallel, sport-specific adaptive profiles of the shoulder complex have been characterised in overhead sports, in which glenohumeral internal rotation deficit (GIRD), increased external rotation and altered external-to-internal rotator strength (ER:IR) ratios have been described. A meta-analysis of 2522 athletes found, on the dominant side, a mean of 9.60° less internal rotation and 6.78° more external rotation, without a significant difference in the total arc of rotation [18]. The clinical significance of these features remains uncertain: subsequent meta-analyses did not show a consistent association between GIRD and upper-limb injury and questioned commonly used diagnostic thresholds [19,20]. Whether this model applies to judo, in which upper-limb exposure involves gripping, pulling, contact with the opponent, throwing and falling rather than repeated overhead throwing, is unknown.

Judo-specific evidence on the shoulder complex is limited and heterogeneous. Impaired joint position sense has been reported in judokas with a history of shoulder injury [21], and in one cohort, selected ER:IR strength ratios were associated with subsequent injury [22]. In a mixed sample of 133 grappling athletes, scapular dyskinesis was observed in 73% of participants, and longer practice was associated with smaller shoulder range of motion (ROM) independently of age, which led the authors to propose the term “combat sports athlete’s shoulder” [23]. Other studies have described the functional shoulder profile of elite athletes [24], rotator asymmetry in female judokas [25] and ROM values in youth categories [26]. These studies differ in population, measurement protocol and construct, and most included small samples.

Previous systematic reviews have addressed the overall injury profile of judo without a detailed analysis of the shoulder girdle [8,27], or shoulder adaptations and risk factors in overhead sports without considering grappling sports [18,19,20]. Consequently, the epidemiology of shoulder girdle injuries and the structural and functional characteristics of the shoulder complex in judokas have not been synthesised together. It therefore remains unclear what proportion of recorded judo injuries involves the shoulder girdle, which injury types and mechanisms predominate, and whether the available studies indicate a reproducible shoulder-complex profile in judokas.

This systematic review aimed to synthesise the available evidence on the epidemiology of shoulder girdle injuries in judokas, including their frequency, anatomical profile, type and mechanism (Q1), and on the structural and functional characteristics of the shoulder complex in judokas, including whether these characteristics are consistent with a sport-specific adaptive profile (Q2).

## 2. Materials and Methods

This review was conducted in accordance with the Preferred Reporting Items for Systematic Reviews and Meta-Analyses (PRISMA 2020) statement [28], and the synthesis was reported according to the Synthesis Without Meta-analysis (SWiM) guideline [29]. Methods followed the Cochrane Handbook for Systematic Reviews of Interventions [30] and, for observational studies reporting prevalence data, the methodological guidance of the Joanna Briggs Institute (JBI) [31]. The protocol was registered in PROSPERO (CRD420261498613). The completed PRISMA 2020 checklist is provided in Table S1. Generative artificial intelligence (Gemini 3.1 Pro; Google LLC, Mountain View, CA, USA) was used only for translation and grammatical correction of the manuscript text.

### 2.1. Eligibility Criteria

Eligibility criteria were defined using the PICOS framework (Table 1). The review addressed two questions: (Q1) what are the frequency, anatomical profile, type and mechanism of shoulder girdle injuries in judokas; and (Q2) what structural and functional characteristics of the shoulder complex are observed in judokas, and are they consistent with a sport-specific adaptive profile? For Q1, a comparator was not required because the denominator of the primary outcome was the total number of recorded injuries or athlete exposure; descriptive surveillance studies without a control group were therefore eligible. For Q2, at least one comparator was required: non-athletes, athletes from other sports, the contralateral limb, normative or reference values, judokas with versus without subsequent injury, or competitive level. Studies of treatment and return to sport after glenohumeral stabilisation, throw kinematics and cross-sectional comparisons of judokas with and without previous shoulder injury (in which the measured feature may result from the injury) were used only as context. Systematic reviews were screened for additional studies but were not a source of primary data.

### 2.2. Information Sources and Search Strategy

PubMed, Web of Science (WoS), Scopus and EBSCOhost (SPORTDiscus, Rehabilitation & Sports Medicine Source, Health Source: Nursing/Academic Edition and OpenDissertations) were searched without restrictions on publication year or document type. The electronic search was supplemented by screening the reference lists of the included studies and of systematic reviews identified during screening. Full search strings for each database are provided in Appendix A.

The search covered records up to 7 August 2026 and was performed by one author (K.K.). The strategy combined a population block and an anatomical-region block with the AND operator, and terms within each block were combined with OR. The population block comprised MeSH descriptors and terms for the discipline (judo*, judoka*, judoist*, judogi*, Kodokan), its technical vocabulary (ukemi, randori, kumi-kata, uchi-komi) and throwing techniques (seoi-nage, tai-otoshi, uchi-mata, harai-goshi, o-soto-gari, tomoe-nage, sode-tsurikomi-goshi, morote-seoi-nage). Japanese terms were included because some reports index techniques without naming the discipline in the title or abstract. In PubMed, three searches were run: the two-block search described above; a population-only search to identify surveillance studies reporting the anatomical distribution of all injuries without mentioning the shoulder in the abstract; and a search for studies of other grappling sports that included a judo subgroup. This structure reflected the definition of the primary outcome, which requires the full anatomical distribution of injuries; restricting the search to shoulder terms could have missed studies reporting shoulder injuries only within a broader injury profile. The strategies for WoS, Scopus and EBSCOhost were adapted to the syntax and search fields of each database while preserving the structure of the primary query.

### 2.3. Selection Process

Records were deduplicated in EndNote 2025.3.1 (Clarivate, London, UK). Two authors (K.K. and W.B.) independently screened records against operationalised eligibility criteria; disagreements were resolved by a third reviewer (T.A.), who is not an author of this review and acted solely as an independent content expert. Screening followed two complementary routes: records containing shoulder girdle terms in the title or abstract, and general judo injury studies without shoulder terms, which were assessed in full text to determine whether they reported an anatomical distribution from which shoulder girdle data could be extracted. Reasons for exclusion at the full-text stage were recorded for each report (Section 3.1). Following PRISMA 2020 terminology [28], we distinguished reports from studies. Publications presenting the same dataset and analysis were treated as multiple reports of one study, and the most complete report was used for the primary outcome. Publications based on the same sample but differing in protocol, follow-up or endpoint were treated as separate studies, without being regarded as independent replication; their participants were counted once.

### 2.4. Data Extraction

Two authors (K.K. and W.B.) independently extracted data using a structured form developed a priori and piloted on three randomly selected reports; disagreements were resolved by the third reviewer (T.A.). Extracted items included bibliographic data, country and setting, study design, data source, sample size and characteristics (sex, age, competitive level, training experience and weight category), injury definition, exposure denominator, recording period, measurement protocol, outcome data with measures of dispersion, and confounders considered in the analysis. When data were incomplete or presented only graphically, the corresponding author was contacted; if no response was received after two messages sent two weeks apart, the report was excluded.

### 2.5. Outcomes and Definitions

For Q1, the primary outcome was the proportion of shoulder girdle injuries among all recorded injuries, which is not a measure of incidence or prevalence. Shoulder girdle injury was defined a priori as an injury of the glenohumeral joint and its periarticular soft tissues, the AC or sternoclavicular joint, or the clavicle, consistent with the shoulder body area of the International Olympic Committee (IOC) consensus [32]. Clavicle or AC joint injuries were added to shoulder injuries only when reported as separate categories, so that no injury was counted twice; component values are shown in Section 3.4.1. Secondary outcomes were incidence per 1000 athlete-exposures or 1000 h of exposure, injury type, injury mechanism and combat phase, severity expressed as time loss, and differences by sex, age and competitive level. For Q2, the primary outcomes were internal rotation (IR) and external rotation (ER) peak torque, the ER:IR ratio and inter-limb differences; secondary outcomes were glenohumeral ROM, scapular position and dyskinesis, posture of the shoulder girdle region, proprioception, and muscle and tendon morphology. Outcomes for which no eligible study was identified are reported as evidence gaps. When results were reported for several time points or subgroups, data for the whole sample were used in the main synthesis, and subgroup data were examined as potential sources of heterogeneity.

### 2.6. Risk of Bias Assessment

The risk-of-bias tool was selected according to study design rather than research question because the same studies could contribute different types of data. Studies reporting injury distributions without comparing exposure groups were assessed with the JBI Critical Appraisal Checklist for Studies Reporting Prevalence Data [31] (items P1–P9). Cross-sectional studies estimating the association between judo practice and a shoulder-complex characteristic were assessed with the revised JBI tool for analytical cross-sectional studies [33] (items C1–C8). Studies estimating the effect of an exposure with a comparator and follow-up were assessed with the Risk Of Bias In Non-randomised Studies of Interventions (ROBINS-I) tool [34] across seven domains: confounding (D1), selection of participants (D2), classification of exposures (D3), deviations from intended exposures (D4), missing data (D5), measurement of outcomes (D6) and selection of the reported result (D7). Overall JBI ratings followed explicit rules (footnotes to the risk-of-bias tables in Section 3.3), and the overall ROBINS-I judgement corresponded to the most severe domain-level judgement [34]. Reports sharing a sample were appraised separately, but each sample contributed once to the GRADE assessment. Two reviewers assessed the risk of bias independently, and disagreements were resolved by a third reviewer. The judgements were used to weight the evidence in the narrative synthesis and to inform GRADE; no study was excluded on the basis of risk of bias alone.

### 2.7. Effect Measures

For Q1, the proportion of shoulder girdle injuries among all recorded injuries was used as the common measure because exposure-based incidence rates were reported in only a few studies. For secondary outcomes, the measures reported in the primary studies were retained, including incidence rates with confidence intervals (CI), odds ratios (OR), percentages, and means with standard deviations. For Q2, the measures reported in the primary studies were used: peak torque in newton-metres or normalised to body mass, the ER:IR ratio (%) and ROM (degrees). Owing to heterogeneous protocols, values were neither converted to a common scale nor expressed as standardised mean differences.

### 2.8. Synthesis Methods

Studies were grouped by outcome and, for the primary Q1 outcome, by exposure context: surveillance limited to competition, recording of training and competition within a defined period, and recording over the entire period of practice (career). For Q2, studies were grouped by construct: rotator strength and ER:IR ratio, inter-limb asymmetry, and ROM, scapular position and posture. Grouping decisions were based on study characteristics before the numerical results were examined.

Random-effects meta-analyses of proportions (Q1) and of standardised mean differences (Q2), with subgroup analyses and meta-regression, were planned but abandoned after data extraction and before the results were interpreted. For Q1, injury definitions and ascertainment methods were not comparable across studies. The denominator was recorded injuries; neither the number of athletes nor the amount of exposure provided a common denominator. Clustering of multiple injuries within the same athlete was not reported, and one survey contributed 72.5% of all injuries [12]. Subgroup analyses and meta-regression were not feasible because individual subgroups included three to six studies, moderators were collinear, and 11 studies were too few for meta-regression [30]. For Q2, only one study included a non-athlete control group. Furthermore, studies differed in contraction mode, angular velocity, test position, and normalisation, and the within-subject correlations required to calculate paired effect sizes were unavailable.

A narrative synthesis with structured tabulation was therefore conducted in accordance with SWiM [29]. The primary Q1 outcome was summarised descriptively overall and by exposure context and ascertainment method, with sensitivity analyses restricted to injuries reported as “shoulder” and excluding reports at high risk of bias. Secondary outcomes were synthesised narratively, giving the greatest weight to prospective studies with medical diagnosis and an exposure denominator and the least to retrospective self-report questionnaires.

### 2.9. Reporting Bias Assessment

Funnel plots and Egger’s test were not applicable in the absence of a pooled estimate. Reporting bias was assessed qualitatively by considering the availability of prospectively registered protocols of the included studies, the restriction to peer-reviewed publications and the distribution of sample sizes within each outcome domain, including the overrepresentation of small samples.

### 2.10. Certainty Assessment

The certainty of evidence was assessed with the Grading of Recommendations, Assessment, Development and Evaluation (GRADE) approach [35] for each outcome across risk of bias, inconsistency, indirectness, imprecision and publication bias, starting at low certainty for observational evidence. No outcome was upgraded: the rank of the shoulder among injury locations is not an effect size, and no comparative effect estimate allowed the upgrading criteria to be applied [36]. Imprecision was judged from the numbers of participants and events, publication bias could not be assessed formally, and data not specific to shoulder girdle injuries were downgraded for indirectness. As a sensitivity check, the descriptive primary Q1 outcome was also rated with the GRADE approach for overall prognosis, in which observational studies start at high certainty [37]. Two reviewers rated certainty independently, and disagreements were resolved by a third reviewer.

### 2.11. Deviations from the Protocol

The planned meta-analyses, subgroup analyses and meta-regressions were not performed, and the reporting of the synthesis was supplemented with SWiM. The stratified and sensitivity analyses of the primary Q1 outcome and the prognostic GRADE rating were added during peer review.

## 3. Results

### 3.1. Study Selection

**Figure 1:**
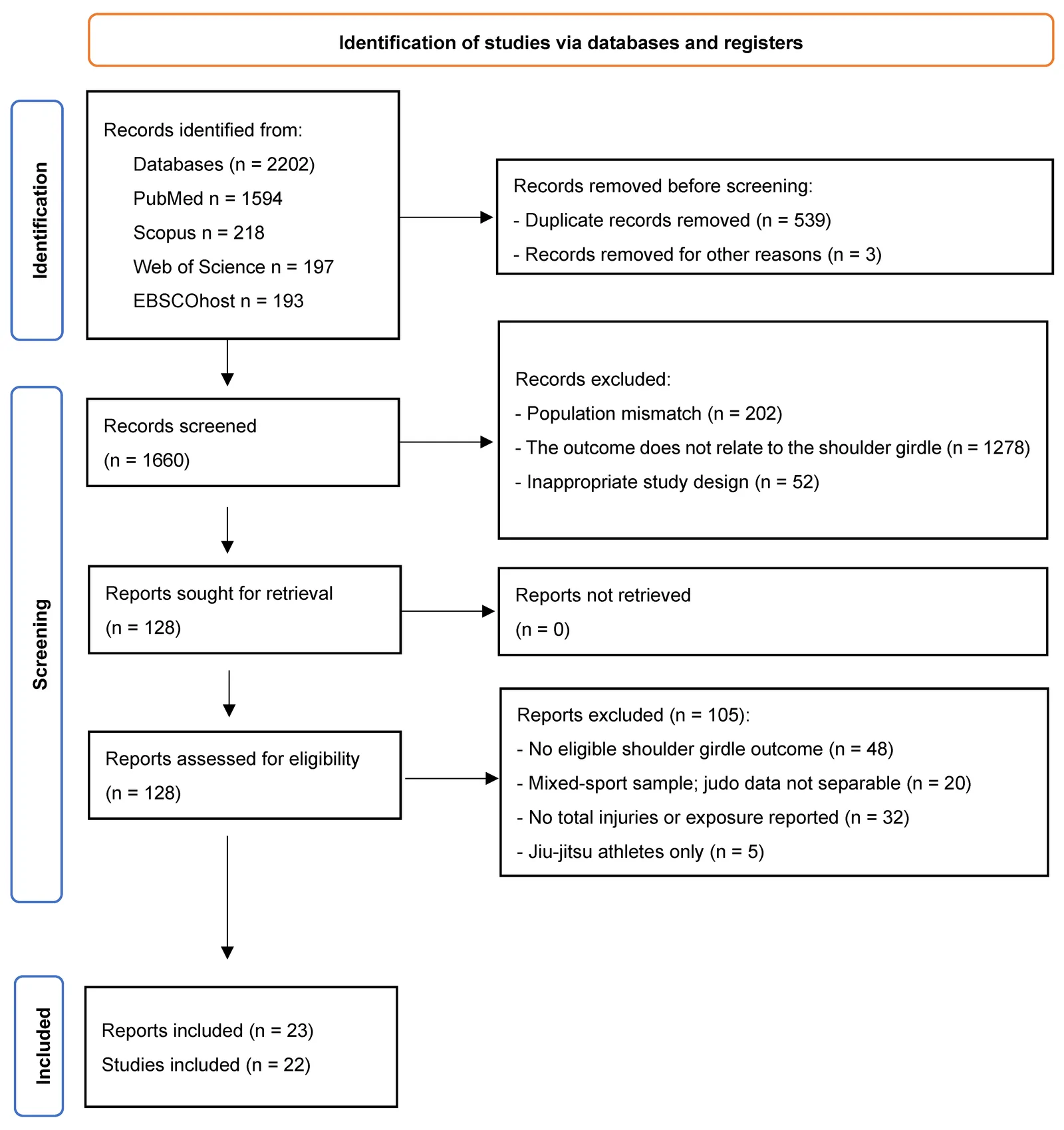
PRISMA 2020 flow diagram.

The searches identified 2202 records: PubMed/MEDLINE (*n* = 1594), Web of Science (*n* = 197), Scopus (*n* = 218) and EBSCOhost (*n* = 193). After removal of 539 duplicates and 3 records for other reasons, 1660 records were screened by title and abstract, and 1532 were excluded (outcome not related to the shoulder girdle, *n* = 1278; population outside the scope, *n* = 202; ineligible study or publication type, *n* = 52). All 128 reports sought for retrieval were assessed in full text, and 105 were excluded: no eligible shoulder girdle outcome (*n* = 48; e.g., a reliability study in physiotherapy students [38], a report without anatomical distribution [39] and five reports used as context [3,4,16,17,21]); mixed-sport samples without separable judo data (*n* = 20; e.g., [23,40]); no total injuries or exposure reported (*n* = 32); and jiu-jitsu athletes only (*n* = 5). The selection process is summarised in Figure 1.

Twenty-three reports of 22 studies (21 independent samples) were included. The reports by Błach et al. from 2021 [15] and 2024 [41] describe one European Judo Union (EJU) registry study; the 2024 report contributed only combat-phase data. The reports by Mian Darbandi et al. [22,42] analysed the same cohort of 53 judokas with different protocols, follow-up periods and endpoints and were treated as two studies of one sample.

### 3.2. Characteristics of Included Studies

The included studies were published between 2006 and 2026 and were conducted in 13 countries, most frequently Brazil, followed by Germany, France and Poland. Thirteen reports (12 studies) addressed Q1, and ten reports (10 studies; nine independent samples) addressed Q2 (Table 2 and Section 3.5). The Q1 studies included approximately 351,000 participants and 15,616 recorded injuries; a single registry accounted for 90% of the participants [10]. The Q2 studies included 405 judokas and 59 control participants, with a median sample size of 36 (range 11–137). This imbalance between the two domains is a key observation of the review and limits the certainty of the conclusions in both. The populations covered the full range of age and competitive level: 8-year-old boys starting judo [43], U18 and U21 athletes [26], university athletes [14,44], national-team and international elite athletes [11,13,22,24] and veterans aged 30–65 years [45]. Female athletes were included in 15 studies, and sex-stratified shoulder girdle data were available from seven Q1 studies (Section 3.4.5).

The studies in Table 2 were grouped by exposure context. Two reports originated from a single EJU registry study; only the 2021 report contributed data on the primary outcome, whereas the 2024 report contributed data on the phase of combat. A total of 699 injuries were recorded, of which 696 had a specified anatomical location. In the study by Barsottini et al., one of the 78 case reports described no injury.

The included studies differed in how the exposure denominator was defined and reported. Only three studies provided data allowing injury incidence to be calculated relative to exposure: 1283 athlete-exposures recorded during three tournaments [46], 975 h of training per athlete per year in the Korean national team [11], and the numbers of athlete-exposures and minutes of combat during the U23 European Championships [13]. In the French registry, injury incidence was expressed relative to the number of participating athletes (1.1% of 316,203 judokas competing in 421,670 bouts) [10], whereas the EJU registry and the questionnaire studies reported only the number of recorded injuries. Direct comparison of incidence rates is therefore possible only between the three studies with an appropriate exposure denominator, and the proportion of injuries by anatomical location is the only measure applicable across all studies; it was therefore adopted as the primary outcome.

### 3.3. Risk of Bias in Included Studies

**Figure 2:**
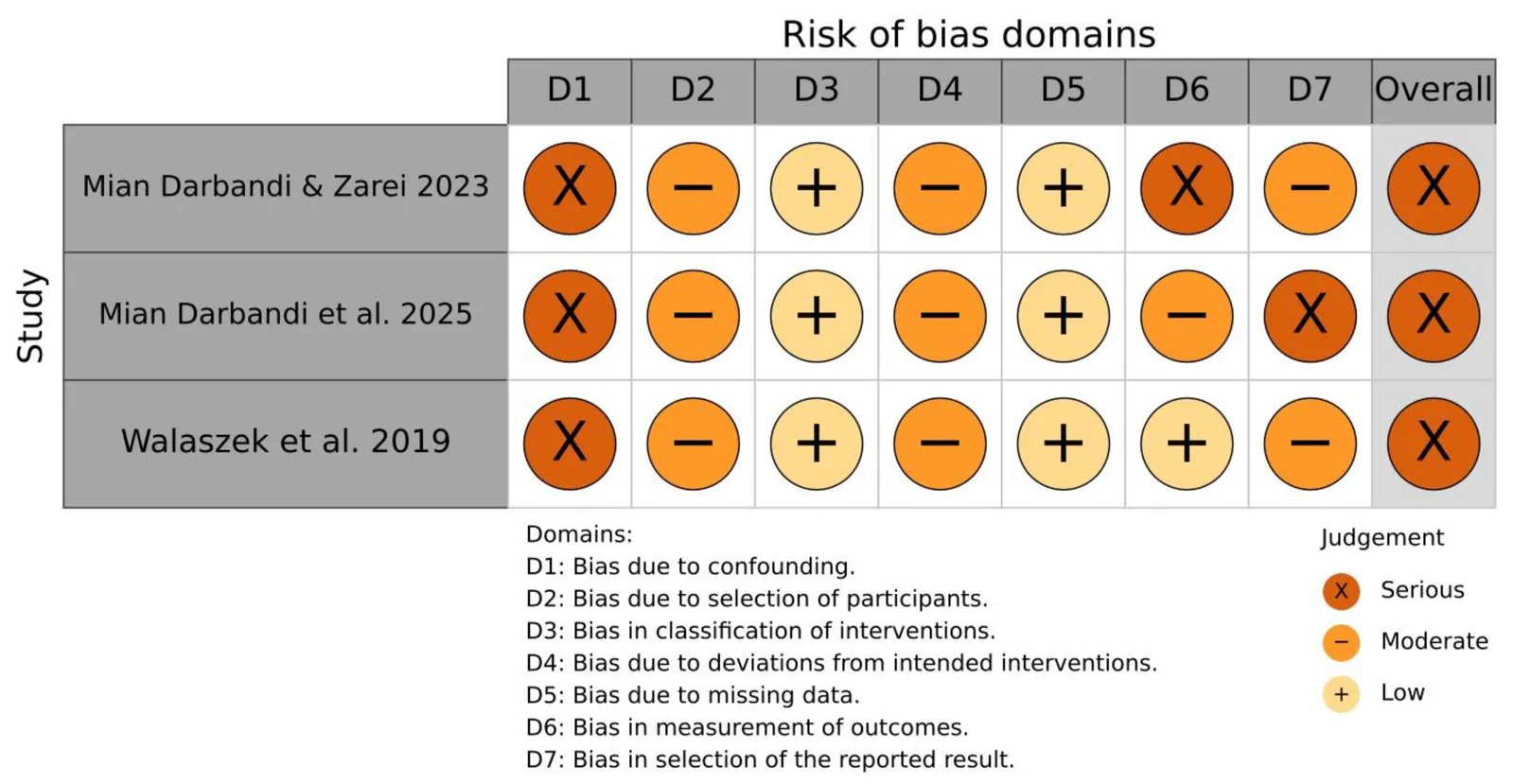
Risk of bias of non-randomised studies (ROBINS-I): domain-level judgements for each study: Mian Darbandi and Zarei 2023 [42], Mian Darbandi et al. 2025 [22] and Walaszek et al. 2019 [43].

**Figure 3:**
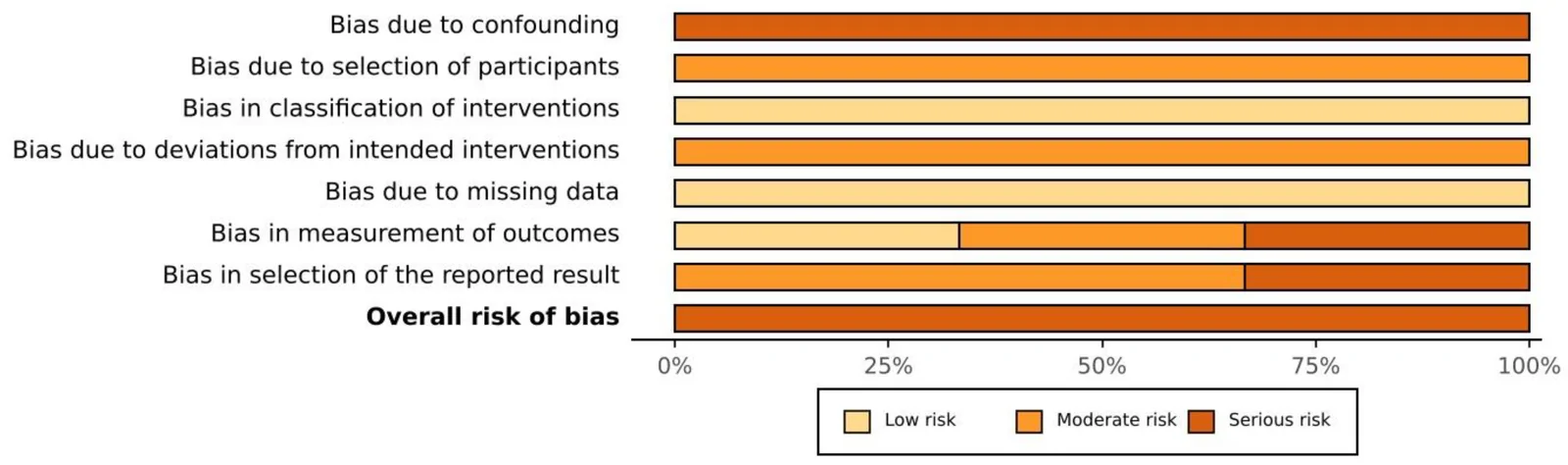
Risk of bias of non-randomised studies (ROBINS-I): proportion of studies with each judgement by domain.

Risk of bias was appraised for all 23 reports: 13 with the JBI prevalence checklist, seven with the JBI analytical cross-sectional tool and three with ROBINS-I (Table 3 and Table 4; Figure 2 and Figure 3). Of the 20 reports appraised with JBI tools, four were rated as being at low, 10 at moderate and six at high risk of bias; all three reports appraised with ROBINS-I were at serious risk of bias. Reports sharing a sample (Błach 2021 [15] and 2024 [41]; Mian Darbandi 2023 [42] and 2025 [22]) were appraised separately but contributed only once to the certainty assessment.

#### 3.3.1. JBI Checklist for Studies Reporting Prevalence Data

The sample frame was appropriate in all 13 reports (P1). Valid identification of the condition (P6) was achieved in the seven reports in which injuries were diagnosed or recorded by medical staff [10,11,13,15,41,46,48], and standard, reliable measurement (P7) in six [13,15,41,45,46,48]. In six questionnaire studies, injury data relied exclusively on self-report without independent verification [12,14,44,45,47,49]. Appropriate statistical analysis (P8) was present in four reports [11,12,13,14]; the others reported percentage distributions without the confidence intervals required by the JBI checklist for prevalence estimates. The response rate (P9) was inadequately reported in all questionnaire studies and was not applicable to the two prospective studies with continuous recording. Low risk of bias was assigned to the two prospective studies that combined medical diagnosis with an exposure denominator: the 4-year Korean national-team cohort [11] and the U23 European Championship cohort [13]. The latter, however, recorded only 24 injuries; a low risk of bias therefore does not imply a precise estimate, and this was considered under imprecision in GRADE (Table 3).

**Table 3:** Risk of bias assessment using the JBI Critical Appraisal Checklist for Studies Reporting Prevalence Data.

Study	P1	P2	P3	P4	P5	P6	P7	P8	P9	Overall Assessment
Souza 2006 [47]	Y	Y	N	Y	Y	N	N	N	N	High
Barsottini 2006 [49]	Y	N	N	Y	N	N	N	N	N	High
Green 2007 [46]	Y	Y	Y	Y	N	Y	Y	N	N	Moderate
Pérez-Turpín 2013 [44]	Y	N	N	N	Y	N	N	N	N	High
Kim 2015 [11]	Y	Y	Y	N	Y	Y	N	Y	N/A	Low
Akoto 2018 [12]	Y	N	Y	Y	N	N	N	Y	N	Moderate
Čierna 2019 [13]	Y	Y	N	Y	Y	Y	Y	Y	N/A	Low
Frey 2019 [10]	Y	Y	Y	N	Y	Y	N	N	N	Moderate
Błach 2021 [15]	Y	Y	Y	N	Y	Y	Y	N	N	Moderate
Błach 2024 [41]	Y	Y	Y	N	N	Y	Y	N	N	Moderate
Kinoda 2024 [14]	Y	N	Y	Y	N	N	N	Y	N	Moderate
Carvalho 2025 [45]	Y	N	N	Y	Y	N	Y	N	N	Moderate
Yacoub 2026 [48]	Y	N	N	N	N	Y	Y	N	N	Moderate

P1: Was the sample frame appropriate to address the target population? P2: Were study participants sampled in an appropriate way? P3: Was the sample size adequate? P4: Were the study participants and setting described in detail? P5: Was the data analysis conducted with sufficient coverage of the identified sample? P6: Were valid methods used for the identification of the condition? P7: Was the condition measured in a standard, reliable way for all participants? P8: Was there appropriate statistical analysis? P9: Was the response rate adequate, and if not, was the low response rate managed appropriately? Overall rating: low, at least 70% of applicable items met, including P6; high, neither P6 nor P7 met and neither P3 nor P8 met; moderate, all other combinations. Responses: Y, yes; N, no or unclear; N/A, not applicable.

#### 3.3.2. Revised JBI Tool for Analytical Cross-Sectional Studies

In this group, the two confounding items were decisive. Confounders were identified in three of the seven studies (C5), and strategies to address them were reported in two (C6). Appropriate statistical analysis (C7) was used in two studies; in the others, the main limitations were small samples without power calculations and multiple comparisons without correction, particularly in studies of 11 and 15 participants [25,50], in which negative findings may reflect insufficient statistical power. Two studies met all eight items and were rated as being at low risk of bias: a study of the functional shoulder profile of 61 elite judokas [24] and a study of 137 U18 and U21 judokas [26]. Three studies were rated as being at high risk; the lowest-rated was a postural assessment based exclusively on qualitative observation without blinding, in which anthropometric and training-history data were available for only 37 of the 50 athletes without explanation [51] (Table 4).

**Table 4:** Risk of bias assessment of analytical cross-sectional studies (revised JBI tool).

Study	C1	C2	C3	C4	C5	C6	C7	C8	Overall Assessment
Ruivo 2012 [52]	Y	Y	Y	Y	Y	N	N	Y	Moderate
Marcondes 2019 [53]	Y	Y	Y	Y	N	N	N	Y	Moderate
Yılmaz 2020 [50]	N	Y	N	Y	N	N	N	N	High
Delorme 2023 [24]	Y	Y	Y	Y	Y	Y	Y	Y	Low
Aedo-Muñoz 2024 [25]	N	Y	N	Y	N	N	N	N	High
Madaleno 2021 [26]	Y	Y	Y	Y	Y	Y	Y	Y	Low
Castropil 2014 [51]	N	N	Y	N	N	N	N	N	High

C1: Were the criteria for inclusion in the sample clearly defined? C2: Were objective, standard criteria used for measurement of the condition? C3: Was the exposure measured in a valid and reliable way? C4: Were the outcomes measured in a valid and reliable way? C5: Were confounding factors identified? C6: Were strategies to address confounding factors stated? C7: Was appropriate statistical analysis used? C8: Were the study participants and setting described in detail? Overall rating: low, at least 85% of items met, including C5 and C6; moderate, 50–84% of items met; high, fewer than 50% of items met. Responses: Y, yes; N, no or unclear.

#### 3.3.3. ROBINS-I Assessment of Non-Randomised Studies of Exposure

ROBINS-I was applied to three studies estimating the effect of an exposure with a comparator: two studies of the same cohort of 53 Iranian national-team judokas [22,42] and a study of boys starting judo who were compared with non-training peers and re-examined after one year [43]. Although they share a sample, the two Iranian studies were appraised separately because they differ in measurement protocol, follow-up and endpoint. All three studies were at serious overall risk of bias. In both Iranian studies, confounding (D1) was rated as serious because the analyses were not adjusted for training volume or age. In the 2023 study, the measurement of outcomes (D6) was also rated as serious because the endpoint combined injuries of the entire musculoskeletal system, including the shoulder and knee, which hinders attribution of the events to the assessed shoulder exposure. In the 2025 study, the selection of the reported result (D7) was rated as serious: more than a dozen strength indices were analysed with only 14 events, and cut-off values were derived post hoc without external validation. In the study of children, confounding (D1) was rated as serious because the groups differed at baseline in five of fifteen indices, and biological maturation and extracurricular physical activity were not controlled; participant selection (D2) was rated as being at moderate risk. In all three studies, the classification of exposures (D3) and missing data (D5) raised no concerns (Figure 2 and Figure 3).

### 3.4. Shoulder Girdle Injuries (Q1)

Results were synthesised narratively in accordance with SWiM, with the proportion of shoulder girdle injuries among recorded injuries as the common measure. Studies are listed by exposure context in Table 2 and in ascending order of the common measure in Table 5.

#### 3.4.1. Proportion of Shoulder Girdle Injuries

Eleven studies provided data for the common measure; the French registry did not report an overall anatomical distribution [10]. Shoulder girdle injuries accounted for 10.2–25.5% of recorded injuries (median 17.1%; IQR 13.2–18.5%). The shoulder ranked first or second among anatomical locations in nine of the 11 studies (first in two [12,44]; second in seven, behind the knee in six and behind the lumbar spine in one [11]); in two small competition cohorts it ranked third [46] and fourth [13], but jointly second when clavicle injuries were added. Within the upper limb, the shoulder was the most frequently injured site in nine studies.

Medians were 15.7% (range 13.2–16.7%) for competition-only surveillance (k = 3), 14.7% (10.2–21.8%) for training and competition within a defined period (k = 4) and 18.4% (18.2–25.5%) for career recall (k = 4), and 15.7% (10.2–17.1%) for medical diagnosis (k = 5) versus 18.4% (12.2–25.5%) for self-report (k = 6). Because all career-recall studies used self-report and all competition-only studies used medical diagnosis, the two factors cannot be separated, and the ranges overlapped.

Restricting the analysis to injuries reported as “shoulder” (k = 9) gave a median of 15.6% (9.2–21.8%); the largest change occurred in one survey in which AC joint dislocations and clavicle fractures accounted for nearly half of the shoulder girdle injuries [12], whereas in the other studies, the change did not exceed 4.2 percentage points. Excluding three reports at high risk of bias gave a median of 16.2% (10.2–18.5%). The range of 10.2–25.5% therefore reflects definitions and surveillance methods and is not an estimate of prevalence (Table 5).

**Table 5:** Proportion of shoulder girdle injuries among recorded injuries in individual studies (ascending order) and descriptive summaries.

Study	Total Injuries (*n*)	Shoulder, *n* (%)	Clavicle/AC Joint, *n* (%)	Shoulder Girdle (%)	Rank of the Shoulder Among Anatomical Locations
Kim 2015 [11]	782	80 (10.2) †	†	10.2	2nd (after lumbar spine/lower back)
Kinoda 2024 [14]	581	71 (12.2)	NR	12.2	2nd (after knee)
Green 2007 [46]	53	6 (11.3)	1 (1.9)	13.2	3rd; shoulder girdle jointly 2nd
Błach 2021 [15]	696	109 (15.7)	NR	15.7	2nd (after knee)
Čierna 2019 [13]	24	3 (12.5)	1 (4.2)	16.7	4th; shoulder girdle jointly 2nd
Yacoub 2026 [48]	105	18 (17.1)	NR	17.1	2nd (after knee)
Barsottini 2006 [49]	77	12 (15.6)	2 (2.6)	18.2	2nd (after knee)
Akoto 2018 [12]	8778	811 (9.2) ‡	787 (9.0) ‡	18.2 ‡	Jointly 1st with the knee
Carvalho 2025 [45]	849	157 (18.5)	NR	18.5	2nd (after knee)
Souza 2006 [47]	110	24 (21.8)	NR	21.8	2nd (after knee)
Pérez-Turpín 2013 [44]	47	12 (25.5) †	†	25.5	1st
Frey 2019 [10]	3511	NR	NR	NR	Most frequent site of dislocation
*Descriptive summaries of the shoulder girdle proportion: median (range)*					
All studies (k = 11)				17.1 (10.2–25.5); IQR 13.2–18.5	
Sensitivity analysis: shoulder only (k = 9) §				15.6 (9.2–21.8)	
Competition only (k = 3)				15.7 (13.2–16.7)	
Training and competition, defined period (k = 4)				14.7 (10.2–21.8)	
Career (k = 4)				18.4 (18.2–25.5)	
Medical diagnosis (k = 5)				15.7 (10.2–17.1)	
Self-report (k = 6)				18.4 (12.2–25.5)	
Sensitivity analysis: excluding reports at high risk of bias (k = 8)				16.2 (10.2–18.5)	

Shoulder girdle, shoulder plus separately reported clavicle/AC joint injuries. † Shoulder and clavicle reported as one category. ‡ Calculated from itemised injury types. § Excluding separately reported clavicle/AC joint injuries and studies with a combined category. Rank refers to the shoulder category as reported. AC, acromioclavicular; IQR, interquartile range; k, number of studies; NR, not reported.

#### 3.4.2. Injury Type

Four studies reported diagnosis-level data for shoulder girdle injuries, all indicating a predominance of acute traumatic conditions. In the largest, the 1598 itemised shoulder girdle injuries comprised 531 AC joint dislocations, 518 unspecified shoulder injuries, 293 shoulder dislocations and 256 clavicle fractures [12]. In a Brazilian state-championship sample, complete dislocations and subluxations together accounted for 11 of 24 shoulder injuries (45.8%) [47]. In the French registry, the AC joint was the most frequently sprained structure (376 of 1907 sprains), the shoulder the most frequent site of dislocation and the clavicle the most frequently fractured bone [10]. In the U23 European Championship cohort, two of three shoulder injuries were dislocations [13]. AC joint injuries and glenohumeral dislocation or instability were therefore the most frequently reported diagnoses; the direction of instability was not reported. No study reported overuse rotator cuff conditions separately, which reflects a gap in surveillance rather than evidence of rarity.

#### 3.4.3. Injury Circumstances and Combat Phase

Six studies reported the circumstances of injury, mostly for all anatomical locations combined. Most injuries occurred in standing combat while executing or receiving a throw. Green et al. recorded 34.2 injuries per 1000 athlete-exposures in standing combat versus 4.7 in groundwork; grip fighting was the most frequent situation in men (11.4 per 1000) and being thrown in women (15.7 per 1000) [46]. In a Brazilian sample, 59.1% of injuries occurred while executing a technique and 24.5% while receiving one [47]. In a German elite cohort, 78.1% of injuries occurred during throwing actions, similarly during the opponent’s (40.0%) and the athlete’s own throw (38.1%) [48]. Barsottini et al. most frequently recorded injuries during ippon-seoi-nage (23%) and tai-otoshi (22%), and 42% of training injuries occurred against a heavier partner [49]. Direct contact was the most common mechanism (68.5%) among Japanese university athletes [14]. Without exposure data for individual techniques or situations, these distributions do not indicate risk.

Shoulder-specific data came only from the EJU registry: shoulder/arm injuries increased from five in the first half of the bout to 15 thereafter, and clavicle/AC joint injuries from five to 11, in parallel with all injuries (67 to 177), so their shares were unchanged (7.5% vs. 8.5% and 7.5% vs. 6.2%) and do not support a shoulder-specific fatigue effect [41].

#### 3.4.4. Severity, Time Loss and Return to Sport

After a shoulder dislocation, 28.0% of athletes returned to sport within 3–6 weeks and 19.5% after more than 6 months; the corresponding values were 23.4% and 7.0% after a clavicle fracture and 33.7% and 13.1% after an AC joint dislocation [12]. After a shoulder dislocation, 52.6% of athletes returned to their pre-injury level and 32.4% to a slightly reduced level, compared with 32.2% and 38.8% after an anterior cruciate ligament rupture [12]. In the EJU registry, 33% of shoulder injuries required transfer to hospital, and the shoulder accounted for 26.5% of such serious injuries but 15.7% of all injuries [15]; without comparable severity data for other sites, this does not establish a priority ranking. Among veterans, 22 shoulder and 59 knee surgeries were reported (2.5% and 6.9% of all injuries) [45].

#### 3.4.5. Sex, Age and Competitive Level

Sex-stratified shoulder girdle proportions were higher in men in five [11,14,15,48,49] and in women in two small competition cohorts with no more than four shoulder girdle injuries per sex [13,46]; tested differences were not significant [14,15]. The incidence of shoulder dislocation was higher in men in the French registry (0.05% vs. 0.02%), which the authors attributed to more close-range gripping [10], whereas Akoto et al. found no sex difference for shoulder dislocation, more AC joint dislocations in men and more clavicle fractures in women [12]. Clavicle fractures predominated in children (0.08% vs. 0.03% in adults), AC joint sprains in young adults (0.20%) and shoulder dislocations in young adults and adults (0.07% and 0.06% vs. 0.02–0.03% in younger groups) [10]. A higher competitive level was associated with more AC joint sprains (0.18% vs. 0.09%) [10] and more AC joint and shoulder dislocations [12].

### 3.5. Shoulder-Complex Characteristics (Q2)

Studies were grouped by construct: rotator strength and ER:IR ratio, inter-limb asymmetry, and ROM, scapular position and posture (Table 6). Because contraction modes and test positions differed, the results were not pooled; a pooled estimate across such protocols would be difficult to interpret and might combine non-comparable constructs.

Measurement protocols were heterogeneous. Isokinetic testing used angular velocities of 60, 180 or 300°/s; four studies shared a concentric protocol at 60 and 180°/s but differed in test position, comparator and normalisation [25,50,52,53]. Test positions ranged from 45° of abduction in the scapular plane [52,53] to 90° of abduction in the supine position for ROM assessment [26]. Biodex (Biodex Medical Systems, Shirley, NY, USA) and Cybex HUMAC NORM (Computer Sports Medicine Inc., Stoughton, MA, USA) dynamometers were used, and torque was expressed in absolute units in some studies and normalised to body mass in others. One study measured only isometric strength [24], and only one included eccentric contractions [22]. These differences precluded a joint quantitative synthesis.

#### 3.5.1. Rotator Strength and ER:IR Ratio

The only study with a non-athlete control group found higher torque values in judokas for all parameters [52]. The difference was larger for internal rotation (74.1 versus 55.0 Nm at 60°/s; +34.7%) than for external rotation (45.4 versus 36.4 Nm; +24.6%), resulting in a lower ER:IR ratio in judokas (60.3% versus 66.9%; *p* = 0.025); at 180°/s, the difference was not significant (63.1% versus 68.7%). The ratio in judokas was below the range of 66–75% proposed in the literature as reflecting balanced rotator strength.

A similarly low ratio was observed in Brazilian elite judokas, with dominant-side ER:IR values at 60°/s of 49.4 ± 7.2% in men and 49.1 ± 4.9% in women [53]; comparison with the previous study is limited by differences in test position and normalisation. Both studies are consistent with greater development of internal than external rotator strength in judokas, possibly related to the demands of kumi-kata; however, neither design can establish whether this profile is specific to judo or reflects selection, body size or concurrent resistance training.

Delorme et al. reported higher internal (*p* = 0.03) and external (*p* = 0.005) rotator strength in younger athletes despite poorer upper-limb stability and power [24]. Individual components of shoulder function may therefore differ independently; the cross-sectional design does not permit inferences about their development over time.

#### 3.5.2. Inter-Limb Asymmetry

Three studies found no significant inter-limb differences in rotator strength or ER:IR ratio. In 20 professional judokas, most side-to-side deficits were below 10%, and the ER:IR ratio was similar on both sides [53]. In 15 elite male judokas, no shoulder strength asymmetry was found, and inter-limb asymmetry was observed only at the knee [50]. In 11 elite female judokas, no clear rotator asymmetry was detected with three analytical approaches [25].

In contrast, Delorme et al. observed an inter-limb difference only in athletes who preferred throwing techniques (*p* = 0.01) [24], suggesting that the technical profile may modify asymmetry; this finding does not show that specialisation in unilateral techniques causes asymmetry. The absence of asymmetry in unstratified samples may be consistent with the bilateral nature of many judo actions, but this hypothesis has not been tested directly.

Small samples and the absence of power calculations further limit interpretation; non-significant inter-limb differences should not be interpreted as evidence of symmetry or equivalence in judokas.

#### 3.5.3. Range of Motion, Scapular Position and Posture

Three studies addressed ROM, scapular position or posture. In 137 U18 and U21 judokas, external rotation ROM differed by limb dominance, and both rotation directions differed by sex [26]. The pattern was not consistent across age categories: in the U18 category, female athletes had a smaller external rotation ROM on the non-dominant side, whereas in the U21 category, external rotation ROM was greater on the dominant side across the whole sample.

In a postural assessment of 50 athletes of the Brazilian national team, shoulder-height asymmetry was observed in 70% and scapular winging in 54%, and only one athlete showed none of the assessed postural deviations [51]. Because the comparator was a normative alignment reference rather than a control group, it cannot be determined whether these features are more frequent than in comparable non-judo populations. The assessment was qualitative, and anthropometric and training data were available for only 37 of the 50 athletes.

The only study with an external comparator and repeated measurements followed 8-year-old boys starting judo [43]. The shoulder position index did not differ between the groups at either time point. Scapular indices differed in the anteroposterior direction at baseline (*p* < 0.05) and in the superoinferior direction after one year (*p* < 0.01), with higher values in judokas in both cases. Because one difference was present before training began, the findings cannot be attributed to one year of judo practice, and their generalisability to adult or experienced athletes is limited.

### 3.6. Association of Shoulder-Complex Characteristics with Injury

One prospective cohort provided data relating rotator strength to subsequent injury. In the 2023 study, concentric shoulder rotator strength at 60 and 300°/s was not associated with injury [42]. In the 2025 study, which added eccentric testing, the eccentric ER:IR ratio of the non-dominant side at 60°/s was associated with subsequent upper-limb injury (OR 0.973; 95% CI 0.951–0.996; *p* = 0.024), as was a bilateral eccentric index at the same velocity (OR 0.043; 95% CI 0.002–0.728; *p* = 0.029) [22]. A cut-off of 72.7 was proposed for the non-dominant ER:IR ratio, with a sensitivity of 0.667 and a specificity of 0.643.

This suggests that eccentric testing may add information to concentric testing; whether it predicts injury better would require independent prospective replication with a direct comparison of both methods.

The evidence is weak: 14 events for many candidate predictors, a cut-off derived and tested in the same sample, a sensitivity and specificity of approximately 0.65 and a serious risk of bias [34]. These findings are hypothesis-generating.

### 3.7. Reporting Bias

Statistical assessment of publication bias, such as funnel plots or Egger’s test, was not possible without a meta-analysis; reporting bias was therefore assessed qualitatively.

None of the included studies had a prospectively registered protocol, which precludes verification of selective outcome reporting. In the Q2 domain, seven of the nine independent samples comprised fewer than 60 participants; if small studies with null results remain unpublished, the available literature may overestimate the magnitude of shoulder-complex differences.

Two instances of overlapping data were identified at the full-text stage. Counting the accompanying reports as independent studies would have double-counted the EJU registry and the Iranian cohort and inflated both the number of studies and the weight of these sources in the synthesis.

### 3.8. Certainty of Evidence

The certainty of evidence was very low for all outcomes (Table 7); no outcome was upgraded, and publication bias could not be assessed formally.

For the primary Q1 outcome, certainty was downgraded for risk of bias (predominantly retrospective self-report and heterogeneous definitions) and inconsistency (10.2–25.5%); the high rank of the shoulder was not used for upgrading because it is not an effect size [36]. With the GRADE approach for overall prognosis, this outcome would be of low certainty [37].

Evidence on injury type, severity and modifiers was downgraded for risk of bias (and, for sex differences, inconsistency), and evidence on injury circumstances also for indirectness because it concerned all injuries.

Q2 outcomes were downgraded mainly for imprecision (median sample size 36; no sample-size calculations) and for inconsistency of isokinetic protocols; prognostic evidence was downgraded for very serious risk of bias and imprecision (14 events in one cohort).

## 4. Discussion

The shoulder girdle was consistently among the two most frequently injured locations in judo (10.2–25.5% of recorded injuries), with AC joint injuries and glenohumeral dislocation or instability predominating. Evidence on shoulder-complex characteristics was sparse and inconsistent, and GIRD and tendon morphology have not been studied in judokas. Certainty was very low for all outcomes, mainly because of risk of bias, heterogeneous definitions and small samples.

### 4.1. Frequency and Patterns of Shoulder Girdle Injuries (Q1)

The consistent ranking contrasts with the variable proportions. The range describes the share of recorded injuries, not risk: a higher share may reflect more shoulder injuries or fewer injuries elsewhere. The stratified analyses suggest that it largely reflects how injuries are defined and captured: competition surveillance records acute events requiring medical attention, career recall captures memorable events, and neither captures complaints managed while training continues. In other sports, questionnaire monitoring identified 419 overuse problems of the knee, lower back and shoulder among 313 athletes, compared with 40 time-loss injuries [54]; the absence of overuse rotator cuff conditions in judo studies therefore more likely reflects surveillance methods than rarity. Definitions consistent with the IOC consensus [32] and the updated Oslo Sports Trauma Research Center questionnaires [55] would capture problems that restrict grips, techniques or throwing side without stopping participation.

The predominance of AC joint injuries and dislocations points to acute mechanisms involving falls and contact. However, the data locate injuries only in broad situations, mostly standing combat while throwing or being thrown, without exposure to specific techniques, grips or roles, so situation-specific risk cannot be estimated. In the EJU registry, the share of shoulder injuries did not increase in later phases of the bout, which argues against a shoulder-specific fatigue effect [41]. Biomechanical studies of the thrown athlete’s shoulder [3,4] and of breakfalls [5,6] suggest that throw type and fall control modulate loading, but they were small, laboratory-based and did not assess injuries. Video analyses of shoulder dislocations in rugby [56,57] provide indirect guidance: judo surveillance should record the athlete’s role (tori or uke), the contact with the opponent and the mat, and the phase of the technique.

Clavicle fractures predominated in children and glenohumeral dislocations in young adults and adults [10], so surveillance and prevention should be age-specific. Age, however, is correlated with previous injury, contact intensity and competitive level, and selection may operate: veteran samples include only athletes who remained active, whereas surveillance of healthy elite athletes omits injured ones. Sex differences were small and inconsistent, and data on female judokas remain insufficient [58].

### 4.2. Shoulder-Complex Profile in Judokas (Q2)

The most consistent functional finding was greater internal than external rotator strength. In the only controlled study, judokas were stronger in both directions, and their lower ER:IR ratio reflected proportionally greater internal rotator strength rather than external rotator weakness [52]; the ratio alone cannot distinguish this profile from low strength in both directions. Values also depend on test position [59] and on normalisation to body mass, which attenuated differences between rugby players and controls [60,61]. No included study related kumi-kata exposure to torque or separated judo from resistance training, selection or body size; these features are therefore characteristics observed in judokas rather than demonstrated adaptations.

Inter-limb asymmetry was not consistently observed, but non-significant differences in samples of 11–20 athletes cannot establish symmetry. Handedness may not reflect the preferred throwing side or the hikite and tsurite roles, and asymmetry found only in athletes preferring throwing techniques [24] suggests that averaging may mask side-specific loading. In handball, external rotation weakness and scapular dyskinesis predicted shoulder injury in one cohort [62] but not in a larger one [63], illustrating the poor replicability of single functional markers.

ROM and scapular data do not indicate an overhead-type pattern. In throwers, ROM changes coexist with osseous adaptations [64], for which no data exist in judokas, whose upper limbs work largely in closed kinetic chains. Static shoulder-height asymmetry and scapular winging are not equivalent to dynamic dyskinesis, which itself was not clearly associated with symptoms (OR 0.79; 95% CI 0.33–1.89) [65] and should not be interpreted as pathology in asymptomatic athletes.

The association between eccentric ER:IR ratios and upper-limb injury [22] is the only prognostic signal. With 14 events, an internally derived cut-off and a serious risk of bias, it does not meet the requirements of a stable prediction model [66] and should not guide decisions such as exclusion from training; moreover, a prognostic factor is not necessarily causal. Its prospective validation against simple predictors, such as previous injury and exposure, is a research priority.

### 4.3. Implications for Practice

The consistent ranking of the shoulder girdle and its over-representation among injuries requiring hospital transfer [15] support explicit shoulder girdle surveillance in judo, with separate coding of glenohumeral, AC joint and clavicle injuries, recurrences and gradual-onset complaints. The evidence does not support specific screening, prevention or return-to-sport protocols. A judo-specific prevention programme did not significantly reduce injury prevalence (23% vs. 28%; OR 0.72; 95% CI 0.37–1.39) and did not target shoulder injuries [67]. Evidence from shoulder injury prevention in handball [68,69,70] and from return-to-sport research after shoulder instability [71,72,73,74,75] is indirect and untested in judo; by analogy, return to technical training, randori and competition could be staged, with assessment in both the tori and uke roles.

### 4.4. Limitations and Future Research

The stratified and sensitivity analyses were post hoc and descriptive, and the composite measure required classification decisions that affected the magnitude of the proportion but not the conclusion that the shoulder is among the most frequently injured sites. Restriction to peer-reviewed publications and the absence of registered protocols in the included studies preclude assessment of selective reporting. In the evidence base, large registries did not ensure certainty, retrospective surveys are prone to recall bias, tournament surveillance misses problems not requiring medical care, and functional studies were small, cross-sectional and poorly controlled for previous injury and load; no eligible study assessed GIRD, proprioception or tendon morphology. Evidence from other sports offers hypotheses rather than judo-specific certainty.

Future studies should use prospective surveillance of training and competition with definitions consistent with the IOC consensus [32,55], parallel exposure data (bouts, throwing role and side, training modifications), repeated bilateral shoulder measurements from the start of the season, and prognostic designs with prespecified endpoints and an adequate number of events [66].

## 5. Conclusions

The shoulder girdle is consistently among the most frequently injured locations in judo: across 11 studies, it accounted for 10.2–25.5% of recorded injuries and ranked first or second in 9. This range reflects heterogeneous definitions and surveillance methods, not prevalence. AC joint injuries and glenohumeral dislocation or instability predominated.

In contrast, evidence for a judo-specific shoulder-complex profile is sparse, heterogeneous and of very low certainty: higher internal rotator strength and a lower ER:IR ratio were observed in some samples, inter-limb asymmetry was inconsistent, and GIRD and tendon morphology have not been studied.

The association of eccentric ER:IR ratios with upper-limb injury, observed in a single small cohort at serious risk of bias, warrants prospective validation rather than routine use.

Current evidence supports systematic shoulder girdle surveillance in judo, with standardised definitions and exposure denominators across training and competition but not specific screening, preventive or return-to-sport protocols.

## Figures and Tables

**Table 1:** Eligibility criteria (PICOS framework).

	Q1—Shoulder Girdle Injuries	Q2—Shoulder-Complex Characteristics
Population	Male and female judo athletes of any age and competitive level who were actively training in judo. Mixed-sport samples were eligible only when data for judo athletes could be extracted separately.	As in Q1; additionally, reporting of training experience was required.
Exposure	Exposure to judo training and competition	Judo training (chronic exposure); within-subject comparison of the dominant vs. non-dominant limb.
Comparator	A comparator is not required. Descriptive epidemiological surveillance studies are eligible without a control group; the denominator is the total number of injuries or athlete exposure.	Non-athletes, athletes from other sports, the contralateral limb, normative values, judokas with vs. without subsequent injury, or reference values reported in the literature for postural assessment.
Outcomes	Primary: Proportion of shoulder girdle injuries (shoulder, acromioclavicular and sternoclavicular joints, and clavicle) among all recorded injuries. Secondary: Incidence per 1000 athlete-exposures or 1000 h of exposure; injury type; injury mechanism (ukemi, kumi-kata, throwing phase, tori vs. uke) and combat phase; injury severity and time loss; differences according to sex, age and competitive level.	Primary: Peak internal and external rotation torque, external-to-internal rotation (ER:IR) strength ratio and inter-limb differences. Secondary: Glenohumeral rotational range of motion; scapular position; posture; proprioception; muscle and tendon morphology.
Study design	Cohort studies, registries and competition-based epidemiological surveillance studies, case–control studies and cross-sectional studies; minimum sample size of 10 participants.	Same criteria as in Q1.

**Table 2:** Characteristics of the studies included in the synthesis of shoulder girdle injuries (Q1).

Study (Country)	Design	Athletes (*n*)	Injuries (*n*)	Recording Period	Injury Definition and Ascertainment
*Competition only*					
Green 2007 [46] (GBR)	Prospective competition surveillance	392	53	3 tournaments	Medical attention or inability to continue
Čierna 2019 [13] (SVK)	Prospective cohort	295	24	1 tournament (U23 European Championships)	Medical attention
Frey 2019 [10] (FRA)	Prospective registry	316,203	3511	21 seasons	Intervention of the competition physician
Błach 2021 [15] (EJU*)	Retrospective registry	28,297	699	128 tournaments, 2005–2020	Medical attention or consultation
Błach 2024 [41] (EJU*)	Retrospective registry	26,862	254	As above	As above; verified by a physician
*Training and competition (defined period)*					
Souza 2006 [47] (BRA)	Cross-sectional, retrospective	93	110	12 months	Self-reported
Kim 2015 [11] (KOR)	Prospective cohort	48	782	4 years	Any musculoskeletal symptom (grades I–III); physician examination
Kinoda 2024 [14] (JPN)	Cross-sectional survey	564	581	12 months	Self-reported
Yacoub 2026 [48] (GER)	Prospective registry	200	105	2.5 years	Time loss ≥ 2 days; physician diagnosis
*Career (entire period of practice)*					
Barsottini 2006 [49] (BRA)	Cross-sectional, retrospective	78	77	Career	Self-reported
Pérez-Turpín 2013 [44] (ESP)	Cross-sectional, retrospective	57	47	Career	Self-reported
Akoto 2018 [12] (GER)	Cross-sectional survey	4659	8778	Career	Self-reported; time loss > 3 weeks
Carvalho 2025 [45] (BRA)	Cross-sectional, descriptive	258	849	Career	Self-reported

Abbreviations: EJU*, European Judo Union; U23, under 23 years of age. BRA, Brazil; ESP, Spain; FRA, France; GBR, United Kingdom; GER, Germany; JPN, Japan; KOR, Republic of Korea; SVK, Slovakia.

**Table 6:** Characteristics of the studies included in the synthesis of shoulder-complex characteristics (Q2).

Study (Country)	Judokas, *n* (Sex)	Comparator	Construct and Protocol	Main Finding
*Rotator strength, ER:IR ratio and inter-limb asymmetry*				
Ruivo 2012 [52] (POR)	22 (M)	Non-athletes (*n* = 22)	Isokinetic strength, CON, 60 and 180°/s; 45° abduction in the scapular plane	Higher ER and IR torque; lower ER:IR ratio at 60°/s (60.3% vs. 66.9%)
Marcondes 2019 [53] (BRA)	20 (10 F)	Contralateral limb	Isokinetic strength, CON, 60 and 180°/s; 45° abduction in the scapular plane	No rotator asymmetry; ER:IR ratio approx. 49% at 60°/s
Yılmaz 2020 [50] (TUR)	15 (M)	Contralateral limb	Isokinetic strength, CON, 60 and 180°/s	No rotator asymmetry
Delorme 2023 [24] (FRA)	61 (38 M)	Contralateral limb; subgroups	Isometric GH rotator strength and functional tests	Asymmetry only in athletes preferring throwing techniques
Aedo-Muñoz 2024 [25] (CHI)	11 (F)	Contralateral limb	Isokinetic strength, CON, 60 and 180°/s; isometric strength	No asymmetry (SPM and PCA analyses)
*Prognostic cohort (association with subsequent injury)*				
Mian Darbandi 2023 [42] (IRN) ‡	53 (M)	Athletes with vs. without subsequent injury	Isokinetic strength, CON, 60 and 300°/s; 9-month follow-up	No association between rotator strength and injury
Mian Darbandi 2025 [22] (IRN) ‡	53 (M)	Athletes with vs. without subsequent injury	Isokinetic strength, CON and ECC, 60 and 300°/s	ECC ER:IR ratios associated with upper-limb injury (14 events)
*Range of motion, scapular position and posture*				
Madaleno 2021 [26] (BRA)	137 (F and M)	Contralateral limb; sex	Rotational ROM, inclinometer, 90° abduction	ER ROM associated with limb dominance and sex
Castropil 2014 [51] (BRA)	50 (27 F)	Normative alignment reference (posture grid and plumb line)	Qualitative postural assessment	Shoulder-height asymmetry 70%; scapular winging 54%
Walaszek 2019 [43] (POL)	36 (M, 8 years)	Non-training age-matched peers (*n* = 37)	Scapular position, Moiré photogrammetry; 1-year follow-up	Differences in scapular indices (one present at baseline)

‡ Two studies of one cohort of 53 judokas (participants counted once). CON, concentric; ECC, eccentric; ER, external rotation; ER:IR, external-to-internal rotation strength ratio; F, female; GH, glenohumeral; IR, internal rotation; M, male; PCA, principal component analysis; ROM, range of motion; SPM, statistical parametric mapping. BRA, Brazil; CHI, Chile; FRA, France; IRN, Iran; POL, Poland; POR, Portugal; TUR, Türkiye.

**Table 7:** Certainty of evidence (GRADE).

Outcome	Studies (Reports)	Risk of Bias	Inconsistency	Indirectness	Imprecision	Publication Bias	Participants/Events	Certainty
*Q1. Shoulder girdle injuries*								
Proportion of shoulder girdle injuries among recorded injuries	11 (11)	Serious ^a^	Serious ^b^	Not serious	Not serious	Not assessable	≈34,900 judokas; 12,102 injuries; 2094 shoulder girdle injuries	⊕○○○ Very low ^c^
Type of shoulder girdle injury	4 (4)	Serious	Not serious	Not serious	Not serious	Not assessable	≥2002 shoulder girdle injuries	⊕○○○ Very low
Injury circumstances and combat phase	6 (6)	Serious	Not serious	Serious ^d^	Not serious	Not assessable	1167 injuries (all locations); 36 shoulder girdle injuries with phase data	⊕○○○ Very low
Severity, time loss and return to sport	3 (3)	Serious	Not assessable ^e^	Not serious	Serious	Not assessable	1864 shoulder girdle injuries	⊕○○○ Very low
Modifiers: sex, age and competitive level	9 (9)	Serious	Serious ^f^	Not serious	Not serious	Not assessable	>350,000 judokas	⊕○○○ Very low
*Q2. Shoulder-complex characteristics*								
Rotator strength and ER:IR ratio	7 (7); 6 samples	Serious	Serious	Not serious	Very serious	Not assessable	182 judokas; 22 controls	⊕○○○ Very low
Inter-limb rotator asymmetry	5 (5)	Serious	Serious	Not serious	Very serious	Not assessable	160 judokas	⊕○○○ Very low
Glenohumeral rotational ROM	1 (1)	Not serious	Not assessable	Not serious	Serious	Not assessable	137 judokas	⊕○○○ Very low
Scapular position and shoulder girdle posture	2 (2)	Serious	Not serious	Not serious	Serious	Not assessable	86 judokas; 37 controls	⊕○○○ Very low
Prognostic value of rotator strength indices for injury	2 (2); 1 sample	Very serious	Not assessable	Not serious	Very serious	Not assessable	53 judokas; 14 upper-limb injuries	⊕○○○ Very low

Certainty started at low (observational studies); no outcome was upgraded. ^a^ Predominantly retrospective self-report; heterogeneous definitions. ^b^ Range 10.2–25.5%. ^c^ Low certainty with the GRADE approach for overall prognosis [37]. ^d^ Data concerned all injury locations. ^e^ Single study per severity measure. ^f^ Inconsistent direction of sex differences. Samples shared by two studies were counted once. Not assessable: no pooled estimate or registered protocols. ER:IR, external-to-internal rotation strength ratio; ROM, range of motion.

## Data Availability

The original contributions presented in the study are included in the article; further inquiries can be directed to the corresponding author.

## Supplementary Materials

The following supporting information can be downloaded at: https://www.mdpi.com/article/10.3390/jcm15208020/s1, Table S1: PRISMA 2020 checklist.

## Abbreviations

The following abbreviations are used in this manuscript: AC, acromioclavicular; CI, confidence interval; CON, concentric; ECC, eccentric; EJU, European Judo Union; ER, external rotation; ER:IR, external-to-internal rotation strength ratio; GH, glenohumeral; GIRD, glenohumeral internal rotation deficit; GRADE, Grading of Recommendations, Assessment, Development and Evaluation; IOC, International Olympic Committee; IQR, interquartile range; IR, internal rotation; JBI, Joanna Briggs Institute; NR, not reported; OR, odds ratio; PICOS, Population, Intervention/Exposure, Comparator, Outcomes, Study design; PRISMA, Preferred Reporting Items for Systematic Reviews and Meta-Analyses; ROBINS-I, Risk Of Bias In Non-randomised Studies of Interventions; ROM, range of motion; SWiM, Synthesis Without Meta-analysis; WoS, Web of Science.

## Appendix A. Search Strategies

PubMed

(“Martial Arts”[Mesh:NoExp] OR judo*[tiab] OR judoka*[tiab] OR judoist*[tiab] OR judogi*[tiab] OR kodokan[tiab] OR ukemi[tiab] OR “kumi kata”[tiab] OR kumikata[tiab] OR “uchi komi”[tiab] OR uchikomi[tiab] OR “seoi nage”[tiab] OR seoinage[tiab] OR “tai otoshi”[tiab] OR taiotoshi[tiab] OR “morote seoi nage”[tiab] OR “sode tsurikomi goshi”[tiab] OR “harai goshi”[tiab] OR “uchi mata”[tiab] OR “osoto gari”[tiab] OR “tomoe nage”[tiab] OR randori[tiab]) AND (“Shoulder”[Mesh] OR “Shoulder Joint”[Mesh] OR “Shoulder Pain”[Mesh] OR “Shoulder Dislocation”[Mesh] OR “Shoulder Fractures”[Mesh] OR “Rotator Cuff”[Mesh] OR “Bankart Lesions”[Mesh] OR “Scapula”[Mesh] OR “Acromioclavicular Joint”[Mesh] OR “Joint Instability”[Mesh] OR shoulder[tiab] OR shoulders[tiab] OR glenohumeral[tiab] OR “gleno humeral”[tiab] OR “rotator cuff”[tiab] OR supraspinatus[tiab] OR infraspinatus[tiab] OR subscapularis[tiab] OR “teres minor”[tiab] OR deltoid[tiab] OR scapula[tiab] OR scapular[tiab] OR scapulae[tiab] OR scapulothoracic[tiab] OR acromion[tiab] OR acromial[tiab] OR subacromial[tiab] OR acromioclavicular[tiab] OR sternoclavicular[tiab] OR glenoid[tiab] OR labrum[tiab] OR labral[tiab] OR bankart[tiab] OR “hill sachs”[tiab] OR “slap lesion”[tiab] OR “slap tear”[tiab] OR latarjet[tiab] OR remplissage[tiab] OR “capsular shift”[tiab] OR capsulolabral[tiab] OR “eden hybinette”[tiab] OR bristow[tiab] OR impingement[tiab] OR dyskinesis[tiab] OR dyskinesia[tiab] OR “internal rotation”[tiab] OR “external rotation”[tiab] OR “rotational motion”[tiab] OR “rotational range”[tiab] OR gird[tiab] OR “glenohumeral internal rotation deficit”[tiab] OR “arc of motion”[tiab] OR “er/ir”[tiab] OR “joint position sense”[tiab] OR isokinetic[tiab] OR “peak torque”[tiab])

judo*[tiab] OR judoka*[tiab] OR judoist*[tiab] OR judogi*[tiab] OR kodokan[tiab] OR ukemi[tiab]

(jujitsu[tiab] OR jujutsu[tiab] OR “jiu jitsu”[tiab] OR “ju jitsu”[tiab] OR “brazilian jiu jitsu”[tiab] OR bjj[tiab] OR sambo[tiab] OR wrestling[tiab] OR wrestler*[tiab] OR grappling[tiab] OR “grappling sports”[tiab] OR “combat sport”[tiab] OR “combat sports”[tiab] OR “martial art”[tiab] OR “martial arts”[tiab]) AND (shoulder[tiab] OR glenohumeral[tiab] OR “rotator cuff”[tiab] OR scapular[tiab] OR bankart[tiab] OR “shoulder instability”[tiab] OR “shoulder injur*”[tiab]) AND (judo*[tiab] OR judoka*[tiab])

Scopus

( TITLE-ABS-KEY ( judo* OR judoka* OR judoist* OR judogi* OR kodokan OR ukemi OR randori OR “kumi kata” OR kumikata OR “uchi komi” OR uchikomi OR “seoi nage” OR seoinage OR “tai otoshi” OR taiotoshi OR “morote seoi nage” OR “sode tsurikomi goshi” OR “harai goshi” OR “uchi mata” OR “osoto gari” OR “tomoe nage” ) ) AND ( TITLE-ABS-KEY ( shoulder* OR glenohumeral OR “gleno humeral” OR “rotator cuff” OR supraspinatus OR infraspinatus OR subscapularis OR “teres minor” OR deltoid OR scapula* OR scapulothoracic OR acromion OR acromial OR subacromial OR acromioclavicular OR sternoclavicular OR glenoid OR labrum OR labral OR bankart OR “hill sachs” OR “slap lesion” OR “slap tear” OR latarjet OR remplissage OR “capsular shift” OR capsulolabral OR “eden hybinette” OR bristow OR impingement OR dyskinesis OR dyskinesia OR “internal rotation” OR “external rotation” OR “rotational motion” OR “rotational range” OR gird OR “glenohumeral internal rotation deficit” OR “arc of motion” OR “joint position sense” OR isokinetic OR “peak torque” ) )

Web of Science

TS=(judo* OR judoka* OR judoist* OR judogi* OR kodokan OR ukemi OR randori OR “kumi kata” OR kumikata OR “uchi komi” OR uchikomi OR “seoi nage” OR seoinage OR “tai otoshi” OR taiotoshi OR “morote seoi nage” OR “sode tsurikomi goshi” OR “harai goshi” OR “uchi mata” OR “osoto gari” OR “tomoe nage”) AND TS=(shoulder* OR glenohumeral OR “rotator cuff” OR supraspinatus OR infraspinatus OR subscapularis OR “teres minor” OR deltoid OR scapula* OR scapulothoracic OR acromion OR acromial OR subacromial OR acromioclavicular OR sternoclavicular OR glenoid OR labrum OR labral OR bankart OR “hill sachs” OR “slap lesion” OR latarjet OR remplissage OR “capsular shift” OR capsulolabral OR impingement OR dyskinesis OR dyskinesia OR “internal rotation” OR “external rotation” OR “rotational motion” OR gird OR “arc of motion” OR “joint position sense” OR isokinetic OR “peak torque”)

EbscoHost (SPORTDiscus; Rehabilitation & Sports Medicine Source; Health Source:Nursing/Academic Edition; OpenDissertations)

( TI ( judo* OR judoka* OR judoist* OR judogi* OR kodokan OR ukemi OR randori OR “seoi nage” OR “tai otoshi” OR “uchi mata” OR “harai goshi” OR “kumi kata” OR “uchi komi” ) OR AB ( judo* OR judoka* OR judoist* OR judogi* OR kodokan OR ukemi OR randori OR “seoi nage” OR “tai otoshi” OR “uchi mata” OR “harai goshi” OR “kumi kata” OR “uchi komi” ) OR SU ( judo* OR “martial arts” ) ) AND ( TI ( shoulder* OR glenohumeral OR “rotator cuff” OR supraspinatus OR infraspinatus OR subscapularis OR deltoid OR scapula* OR acromion OR subacromial OR acromioclavicular OR sternoclavicular OR glenoid OR labrum OR labral OR bankart OR latarjet OR impingement OR dyskinesis OR “internal rotation” OR “external rotation” OR isokinetic OR “peak torque” ) OR AB ( shoulder* OR glenohumeral OR “rotator cuff” OR supraspinatus OR infraspinatus OR subscapularis OR deltoid OR scapula* OR acromion OR subacromial OR acromioclavicular OR sternoclavicular OR glenoid OR labrum OR labral OR bankart OR latarjet OR impingement OR dyskinesis OR “internal rotation” OR “external rotation” OR isokinetic OR “peak torque” ) OR SU ( shoulder* OR “rotator cuff” OR “shoulder injuries” ) )
